# Perfusion Computed Tomography May Help in Discriminating Gastrointestinal Tuberculosis and Crohn’s Disease

**DOI:** 10.3390/diagnostics13071255

**Published:** 2023-03-27

**Authors:** Raghav Seth, Pankaj Gupta, Uma Debi, Kaushal Kishore Prasad, Harjeet Singh, Vishal Sharma

**Affiliations:** 1 Department of Radiodiagnosis and Imaging, Postgraduate Institute of Medical Education and Research, Chandigarh 160012, India; raghav_seth@hotmail.com (R.S.); pankajgupta959@gmail.com (P.G.); 2 Department of Gastroenterology, Postgraduate Institute of Medical Education and Research, Chandigarh 160012, India; kaushalkp10@hotmail.com (K.K.P.); docvishalsharma@gmail.com (V.S.); 3 Department of Surgical Gastroenterology, Postgraduate Institute of Medical Education and Research, Chandigarh 160012, India; harjeetsingh1982@gmail.com

**Keywords:** perfusion, tuberculosis, Crohn’s disease, computed tomography

## Abstract

Gastrointestinal tuberculosis (GITB) and Crohn’s disease (CD) are close mimics. This prospective study aimed to evaluate the diagnostic performance of perfusion computed tomography (CT) in differentiating GITB from CD. Consecutive patients with ileocaecal thickening underwent perfusion CT of the ileocaecal region between January 2019 and July 2020. Two radiologists (blinded to the final diagnosis) independently assessed blood flow (BF), blood volume (BV), mean transit time (MTT), and permeability at perfusion CT. These parameters were compared among the patients with GITB as well as active and inactive CD. Receiver operating characteristic curves were utilized for determining the diagnostic performance of perfusion CT. Interclass correlation coefficient and Bland–Altman analysis were performed to compare the observations of the two radiologists. During the study period, 34 patients underwent perfusion CT. Eight patients had diagnoses other than intestinal tuberculosis or CD. Thus, 26 patients (mean age 36 ± 14 years, 18 males) with GITB (*n* = 11), active CD (*n* = 6), and inactive CD (*n* = 9) were evaluated. BF, MTT, and permeability showed significant differences among the groups, while BV did not differ significantly among the groups. BF and permeability had 100% sensitivity and 100% specificity, while MTT had 61.5–100% sensitivity and 70–100% specificity for differentiating GITB from active CD and active from inactive CD. The interclass correlation coefficient for perfusion CT parameters was 0.88–1. Perfusion CT is a novel imaging technique that can improve the diagnostic performance of differentiating tuberculosis from CD.

## 1. Introduction

Crohn’s disease (CD) and gastrointestinal tuberculosis (GITB) are common causes of intestinal diseases. These two conditions are an important diagnostic dilemma in countries where TB is endemic and where inflammatory bowel diseases, including CD, are increasing [1]. CD and GITB are difficult to differentiate owing to overlapping clinical, radiological, endoscopic, and histological features. Both of these diseases have similar clinical features, including abdominal pain, diarrhea, intestinal obstruction, and loss of weight [2]. The imaging findings are also similar with the presence of bowel thickening, mural enhancement and stratification, and lymphadenopathy. Endoscopic findings include ulceration, strictures or narrowing, pseudo-polyps, skip lesions, and so on, with overlap in the types of ulcers between the two conditions. Histology may demonstrate granulomatous pathology in both entities. Caseating necrosis, although specific for GITB, is an infrequent finding [3]. Both of these entities show changes in chronic architectural distortion that are non-specific. Unfortunately, GITB is usually a paucibacillary condition and microbiological tests are positive only in a minority of patients [4]. As differentiation is often not possible, clinicians in endemic regions often start antitubercular therapy (ATT) empirically and assess the response to therapy. Unfortunately, clinical response to empirical ATT can occur even in CD, thus a repeat colonoscopy may be needed to be sure of an early mucosal response. This strategy, apart from being costly, has potential risks, including ATT-induced hepatitis and the progression of CD as a result of the delay in the initiation of CD-specific therapy. Therefore, the differentiation of the two diseases has immense clinical significance, considering the entirely different therapeutic strategies and prognoses.

Imaging plays an essential role in evaluating small bowel and colonic diseases. Conventional imaging techniques (barium studies) have been used to differentiate GITB and CD in the past, but, as they cannot provide information about the extra-luminal features of both diseases, their role in the differentiation of the two is limited [5]. Spectral CT has also been evaluated to assess Crohn’s disease in terms of disease activity. It demonstrated a higher accuracy than conventional CT in predicting intestinal activity [6]. However, its role in the differentiation of gastrointestinal TB and CD has not been studied.

A few recent CT imaging techniques (carbon dioxide enterography and positron emission tomography/CT enterography) have shown encouraging results in the differentiation of the two diseases [7,8]. However, these techniques are not widely available and entail high costs and a lot of time. In addition, a few CT-based predictive models for the differentiation of the two diseases have also been evaluated [6,9,10]. However, they are not commonly used because of the lack of standardization of the variables used in different predictive models. Thus, an accurate and reproducible imaging test to differentiate between the two diseases is required.

Perfusion CT (PCT) is a novel technique used in oncology for diagnosis, prognosis, and treatment response evaluation and has proved to be highly accurate [11,12]. However, most studies assessing bowel perfusion have investigated the large bowel. In contrast, its role in the small bowel is relatively unexplored, with only a single study demonstrating the feasibility of PCT in the small bowel [13].

Furthermore, many studies have evaluated the microvascular environment and mesenteric vasculature in the two diseases. These studies have shown significant differences in the vascularity of GITB and CD [14,15,16,17,18,19]. Thus, based on this hypothesis, we conducted this prospective study to evaluate whether PCT enterography can help differentiate GITB and CD.

## 2. Materials and Methods

The present study was conducted in accordance with the Declaration of Helsinki and ICMR guidelines for ethical human research. The study protocol was approved by the institutional ethics committee. Informed written consent was obtained from all participants prior to inclusion.

**Patient Selection**

Consecutive patients (age > 18 years) presenting to the gastroenterology OPD who were clinically suspected of having GITB or CD and who were diagnosed with ileocaecal thickening were included in the study (study period: January 2019 to July 2020). The clinical pointers towards GITB/CD included one or more of the following: diarrhea, abdominal pain attributable to the small bowel, or sub-acute intestinal obstruction. Ileocaecal thickening was diagnosed on ultrasound, CT, or magnetic resonance imaging. We excluded pregnant females, patients allergic to intravenous or oral contrast, patients with renal insufficiency, and patients refusing to give consent.

**CT Data Acquisition**

The scans were acquired on a multi-detector row CT scanner (Somatom Definition Flash 128 multi-detector row CT scanner, Siemens Healthcare, Erlangen, Germany). A neutral oral contrast agent, polyethylene glycol (1.2 L), was administered over 30 min before the image acquisition. First, a CT scanogram of the abdomen was obtained. Following this, a low-dose non-contrast CT scan from the inferior surface of the liver to the iliac crest was obtained for localizing the ileocaecal junction. A pre-defined volume (measuring 15 cm on the z-axis) was centred at the ileocaecal junction. Then, an intravenous injection of 20 mg of hyoscine butyl bromide was administered. An abdominal band restraint was used to minimize movements of the abdominal wall. Next, an iodinated contrast agent, 40 mL (300 mg I/mL; Omnipaque 300, GE Healthcare, Chicago, IL, USA), was injected, followed by 30 mL of normal saline, both injected at 5.5 mL/s using a pressure injector. The following scanning parameters were used: tube current–exposure time product (mAs) of 90, tube voltage setting (kVp) of 70, pitch of 0.7, and reconstruction slice thickness of 1.5 mm with a reconstruction increment of 1 mm. The low-dose dynamic scan (15 cm) centered on the ileocaecal junction was obtained at 1.5 s intervals, 3 s following the intravenous injection of the contrast. The total duration of the examination was 52.5 s.

**Perfusion CT Analysis**

**Figure 1:**
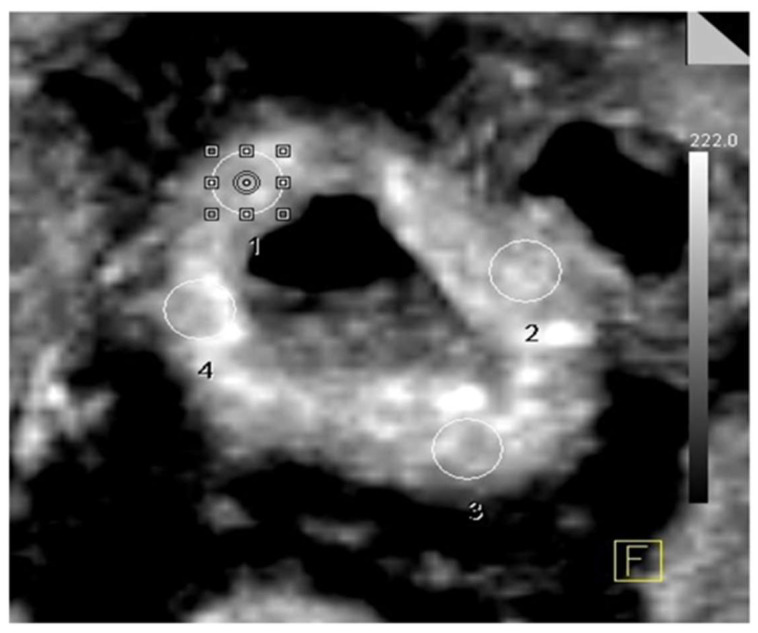
Technique of region of interest (ROI) placement. 1, 2, 3, and 4 represent the ROIs placed on the anterior, lateral, posterior, and medial walls, respectively, on the axial section of the involved bowel loop.

Data analysis was performed using dedicated software (Volume Perfusion CT and Syngo Multimodality Workplace, Siemens Healthcare). The first step consisted of motion correction, which was carried out using the inbuilt motion-correction algorithm and manually excluding the distorted sections owing to the patient’s motion. Then, automatic bone identification and segmentation were performed, followed by placing a region of interest (ROI) on the abdominal aorta proximal to its bifurcation. The application automatically constructed the perfusion maps using the deconvolution pharmacokinetics model. Four ROIs were placed on each axial section of the bowel wall of the involved segment, with a 1 cm interval between each section. The image was magnified to such an extent that the involved bowel cross section covered approximately 25% of the screen. The size of the ROI was decided according to the extent of bowel thickening: an ROI of 0.1 cm^2^ for bowel thickening of less than 5 mm, 0.2 cm^2^ for bowel thickening of 5–10 mm, and 0.3 cm^2^ for bowel thickening of >10 mm. If there was asymmetrical bowel wall thickening, the ROI size was taken according to the maximum thickening present in the respective bowel wall as per the criteria mentioned above. While placing the ROIs on the bowel wall, care was taken to exclude visible vessels and non-enhancing areas within the visualized bowel wall (Figure 1). The ROI size was adjusted so that there were no partial volume effects while sampling.

Four PCT parameters were assessed. These included the following:

**BF**: blood flow [mL/100 mL/min], defined as the flow rate through the vasculature in the tissue of interest; **BV**: blood volume [mL/100 mL], defined as the volume of blood flowing within the vasculature of the tissue of interest; **MTT**: mean transit time [seconds], defined as the average time taken by the contrast medium to travel from the artery to the vein; and **PMB**: permeability [mL/100 mL/min], defined as the total blood flow from plasma to the interstitial space.

The PCT parameters were estimated independently by two radiologists (with eight years and two years of experience in abdominal CT), who were blinded to the final diagnosis. For each patient, each radiologist recorded several values of perfusion parameters, ranging from a minimum of 48 values to a maximum of 160 values, depending on the total length of the thickened bowel segment in the respective patient (a total of 16 values for the perfusion parameters were recorded for each section of bowel wall thickening and subsequent measurements were taken at a distance of 1 cm). Then, the mean of the values was calculated and inter-observer agreement was assessed.

**Reference Standard**

The internationally approved criteria were used for the diagnosis. For the diagnosis of CD, the European Crohn’s and Colitis Organization and European Society of Gastrointestinal and Abdominal Radiology guidelines were used [20]. To evaluate CD activity, we used the Crohn’s Disease Activity Index (CDAI) with a cut-off of 150 to divide active and inactive CD.

For GITB, the INDEX-TB definition was used [21]. If a microbiological test like mycobacterial culture or a standard polymerase-chain-reaction-based test (Xpert MTB/RIF) was positive, a diagnosis of microbiologically confirmed GITB was made. However, if mycobacterial tests were negative or if there was diagnostic confusion, we started a diagnostic trial of ATT. In such patients, a colonoscopy was repeated at two months to assess for mucosal healing. Early mucosal response (ulcer healing at two months after a diagnostic trial of antitubercular therapy), as suggested by the Indian Council of Medical Research Standard Treatment Workflow, was used to confirm the diagnosis of GITB [22,23].

**Statistical Analysis**

The data were coded and recorded in the Microsoft Excel spreadsheet program. Descriptive statistics were elaborated as the mean and standard deviation for continuous variables and proportions and percentages for categorical variables. When comparing two groups, comparisons were made using the Mann–Whitney U-test and the Kruskal–Wallis test was used when comparing more than two groups. For paired data, the Wilcoxon-signed rank test was used. Receiver operating characteristic (ROC) curves were plotted to determine the diagnostic performance and best cut-off for perfusion parameters in predicting a dichotomous outcome. An interclass correlation coefficient and Bland–Altman analyses were performed to compare the observations of the two radiologists. SPSS v. 23.0 (IBM Corp., Armonk, NY, USA) was used for data analysis. A *p*-value of <0.05 was considered statistically significant for all analyses.

## 3. Results

### 3.1. Demographic Data

Over the study period, 34 patients underwent PCT. GITB and CD were diagnosed in 11 and 15 patients, respectively. Out of the CD patients, the number of active and inactive CD cases was 6 and 9, respectively. Other diagnoses (n = 8) included appendicitis (n = 1), amoebic colitis (n = 1), adenocarcinoma of the caecum (n = 1), ulcerative colitis (n = 1), non-specific colitis (n = 3), and complicated enteric fever (n = 1). Thus, we included 26 patients in the analysis. The mean (±SD) age was 36 ± 14 years, 18 males. One CD patient had fistulizing disease. Colonic involvement was observed in three patients with GITB and four patients with CD.

### 3.2. PCT Parameters

**Figure 2:**
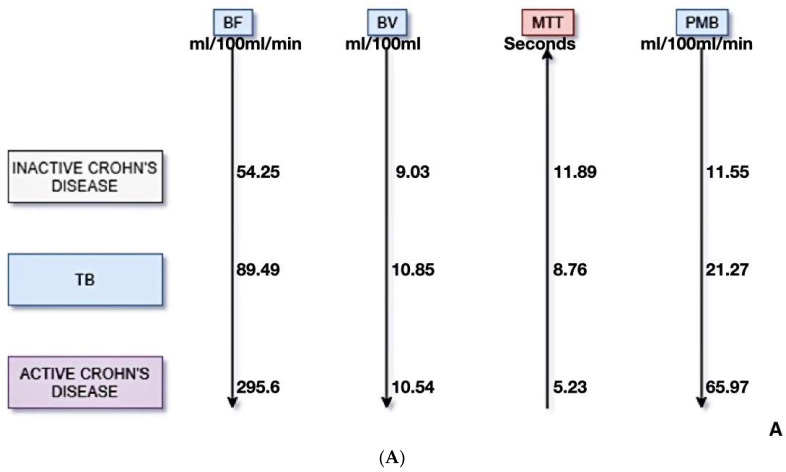

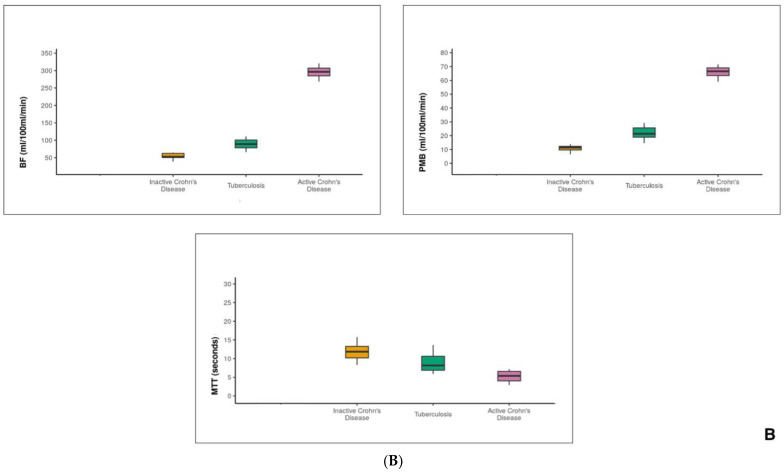
(**A**) Graphical depiction showing the trend followed by the perfusion parameters. (**B**) Corresponding box and whisker plots. There is an increasing gradient in blood flow (BF) and permeability (PMB) from inactive CD to gastrointestinal tuberculosis to active CD. A reverse gradient is noted for mean transit time (MTT). (BF: Blood Flow, BV: Blood Volume, MTT: Mean Transit Time, PMB: Permeability, TB: Tuberculosis).

All PCT scans were diagnostic. The mean values of the perfusion parameters in the inactive CD, GITB, and active CD patients were BF (mL/100 mL/min) of 54.3, 89.5, and 295.6, respectively (*p* = 0.001); BV (mL/100 mL) of 9.03, 10.85, and 10.54, respectively (*p* = 0.47); MTT (seconds) of 11.9, 8.8, and 5.2, respectively (*p* = 0.001); and PMB (mL/100 mL/min) of 11.6, 21.3, and 65.9, respectively (*p* = 0.001) (Table 1, Figure 2).

Upon pair-wise comparison of the perfusion variables between the groups, BF, MTT, and PMB showed statistically significant differences among all of the groups. BV did not show a statistically significant difference between the groups.

### 3.3. Diagnostic Performance of PCT

**Figure 3:**
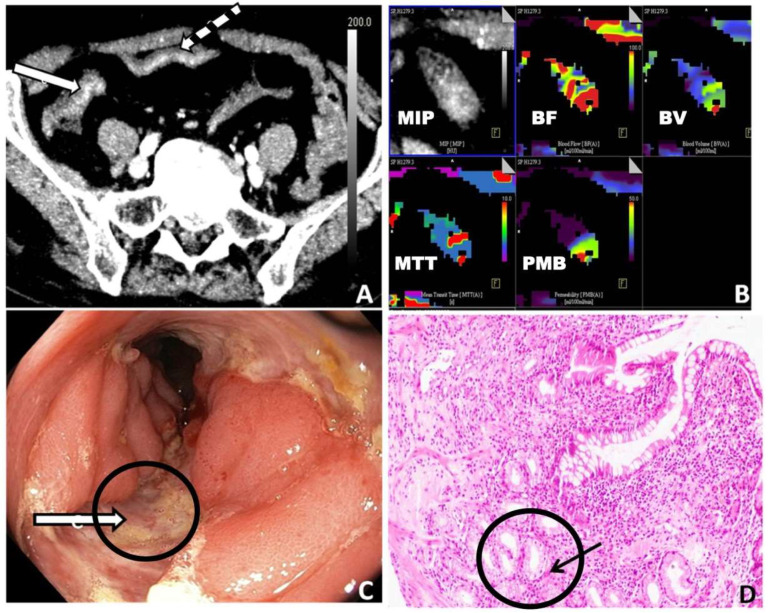
Case of Crohn’s disease. (**A**) Axial section from the perfusion CT base image showing enhancing mural thickening of the terminal ileum (white arrow with black striations) and caecum (white arrow). (**B**) Corresponding color-coded perfusion maps showing MIP, BF, BV, MTT, and PMB images. (**C**) Colonoscopic image of the same patient showing deep serpiginous ulcers (marked with a circle and an arrow). (**D**) Histopathological findings of the same patient: ileal mucosal biopsy shows an excess of mononuclear inflammatory cells admixed with eosinophils in the lamina propria along with foci of pyloric metaplasia (marked with a circle and an arrow) (H&E X200) (MIP: maximum intensity projection; BF: blood flow; BV: blood volume; MTT: mean transit time; PMB: permeability; H&E: haematoxylin and eosin).

**Figure 4:**
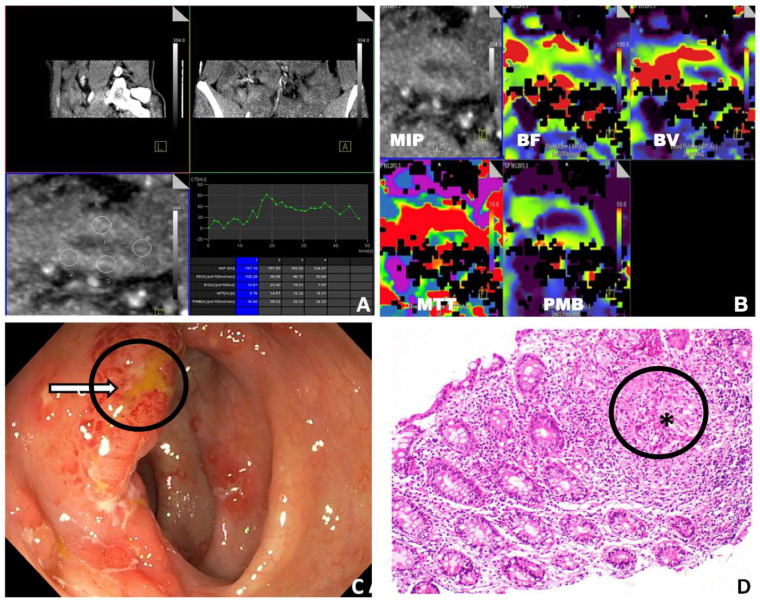
Case of gastrointestinal tuberculosis. (**A**) Post-processing perfusion CT images showing ROI placement and values of perfusion parameters. (**B**) Corresponding color-coded perfusion maps showing MIP, BF, BV, MTT, and PMB images. (**C**) Colonoscopic images of the same patient showing an oedematous and hyperaemic ileocaecal valve with a small ulcer (marked with a circle and an arrow). (**D**) Histopathological findings of the same patient: ileocaecal mucosal biopsy shows a non-caseating granuloma (marked with a circle and *) in the lamina propria (H&E X200) (MIP: maximum intensity projection; BF: blood flow; BV: blood volume; MTT: mean transit time; PMB: permeability; H&E: haematoxylin and eosin).

The highest sensitivity, specificity, and AUC were achieved with BF and PMB (Table 2). The diagnostic performance for differentiating GITB from the entire CD group (both active and inactive) and GITB from the inactive CD was lower than that for other groups (Table 2). BV showed the lowest diagnostic performance. Figure 3 and Figure 4 demonstrate the perfusion maps of cases of CD and GITB, respectively.

### 3.4. Interobserver Agreement

The interobserver agreement between the two radiologists for evaluating the perfusion parameters was excellent. The interclass correlation coefficients were 1.0, 0.88, 0.91, and 0.99 for BF, BV, MTT, and PMB, respectively.

### 3.5. Radiation Dose

The mean CTDI vol and DLP were 32.52 ± 6.04 mGy and 407.42 ± 75.81 mGy.cm, respectively.

## 4. Discussion

In this prospective study, we evaluated PCT parameters in three groups: GITB, active CD, and inactive CD. We found that three out of the four perfusion parameters (BF, MTT, and PMB) showed a statistically significant difference. The PCT parameters had high sensitivity and specificity for diagnosing CD and GITB. We also found an excellent correlation between the two observers, indicating that the modality is reproducible. This study emphasizes the potential role of PCT in the differentiation of GITB and CD and is a first-of-its-kind evaluation of perfusion CT in solving this important clinical dilemma.

Correctly differentiating between the two diseases is essential, as an incorrect diagnosis has profound implications [1]. For example, if GITB is wrongly diagnosed as CD and immunosuppressive drugs are given, it will result in a flare-up and dissemination of TB throughout the body, whereas, if a patient with CD is wrongly diagnosed as having GITB, it will not only cause a delay in the initiation of the correct therapy but also lead to unnecessary exposure of the patient to antitubercular therapy, which may cause multiple harmful effects like a liver injury. Many of the world’s most populous regions (including India and China) grapple with this problem—they are endemic for tuberculosis, while inflammatory bowel diseases are rising in these regions. It is now well recognized that a diagnostic delay in Crohn’s disease, also attributed to the administration of ATT, may result in increased surgical requirements. Interestingly, administration of ATT in CD has also been reported to result in the conversion of the inflammatory phenotype to the stricturing phenotype, suggesting that unnecessary ATT in CD should be avoided where possible.

Routine laboratory tests, including total leukocyte count, differential leukocyte count, low hemoglobin, raised erythrocyte sedimentation rate, raised C-reactive protein, and raised faecal calprotectin levels, can be seen in the active phases of both diseases [24,25]. Colonoscopy can help in the differentiation of the two diseases in a subset of cases, but there is a significant overlap in the colonoscopic features of GITB and CD, and no single finding is unique for the diagnosis of either of the two conditions. As far as histopathological analysis is concerned, both CD and GITB are chronic granulomatous diseases, and there are minimal histopathological differences between them. If the granulomas are associated with caseous necrosis or the stain for AFB is positive, then the biopsy is diagnostic for GITB. However, the yield of sampling is very poor, thus histopathology is diagnostic in very few cases (<30%) [3]. In addition, sampling bias is also of great concern in biopsy assessment and has a significant impact on the diagnostic results. Furthermore, there is a lack of feasible methods to carry out such sampling bias studies, as it is not feasible to take numerous needle biopsies from the same patient just to support such research [26].

In recent times, there has been an increasing emphasis on molecular diagnosis of TB. According to a report on the use of primers (IS 6110) for the diagnosis of ITB, the sensitivity and specificity of the test were 46% and 95%, respectively [27]. This means that, although a positive PCR (polymerase chain reaction) can support the diagnosis of GITB, a negative PCR cannot exclude GITB. GeneXpert TB (“Xpert MTB/RIF”) is a cartridge-based nucleic acid amplification test that can not only identify MTB DNA but also detect resistance to Rifampicin. It is useful in pulmonary TB and TB of the lymph nodes, but the sensitivity of this test for GITB is very low (around 23%) [28].

Imaging modalities like barium studies have been used to differentiate GITB and CD in the past. Still, as they cannot provide information about the extra-luminal features, their role in differentiating between the two diseases is limited [29]. Routine abdominal CT fails to distend the small bowel, thus it is challenging to evaluate the mural changes, including enhancement characteristics associated with GITB or CD. CT enterography features may help differentiate GITB from CD [5]. A long segment ileal involvement with more than three segments involved and skip areas with engorged vasa recta favor the diagnosis of CD, whereas multi-segmental symmetrical mural thickening of the ileocaecal area and necrotic lymph nodes supports the diagnosis of GITB [5,30,31]. However, the precise differentiation between the two entities is only possible in some patients.

Spectral CT has been utilized to assess CD activity and severity [6]. In one study, spectral CT demonstrated a higher accuracy (99.6% versus 94.7%), sensitivity (99.1% versus 93.4%), and specificity (99.9% versus 94.4%) than conventional CT in predicting CD activity. However, its role in intestinal TB and CD differentiation has not been studied.

Therefore, despite previous attempts at imaging to differentiate between the two entities, none of them are accurate enough to distinguish the two diseases. Therefore, there is a need for an accurate, objective, and clinically applicable method for the differentiation of GITB and CD.

Furthermore, in cases of CD, accurate differentiation between active and inactive CD cases is also very important. In CD, both inflammatory and fibrotic types of strictures can be observed. The strictures can be detected by other radiological modalities like CT enterography, MR enterography, and ultrasonography, but determining whether the stricture is inflammatory or fibrotic is challenging [30]. It is important to make this distinction, as the management of the disease relies on it. Active CD cases can be managed well with medical management. However, for chronic or inactive CD patients who develop fibrotic strictures, no therapeutic agent is available that can inhibit or reverse the fibrosis. The only management strategy available for these cases is endoscopic dilatation. Therefore, differentiation between the two types of CD is indispensable. In such a scenario, perfusion CT, being highly sensitive, specific, and reproducible, can serve as a great tool to help differentiate active and inactive CD.

The results of our study (values of the perfusion parameters in CD and GITB) can be explained as follows.

Early inflammation in CD is characterized by increased perfusion, whereas, paradoxically, in chronically inflamed tissues, there is a decrease in the regional blood flow, leading to prolonged ischemia and microinfarction [15,16,17,18,19]. Studies that have evaluated vascular changes in GITB have revealed differing results [14,15,16,17,18,19]. Some studies have reported thrombosis of medium and large vessels and endarteritis of submucosal vessels, leading to mucosal ulceration, whereas others have reported mildly increased vascularity in TB ulcers. Thus, active CD shows the highest BF, BV, and PMB and the shortest MTT. On the other hand, inactive CD (showing chronic inflammation and fibrinoid vascular occlusion) results in lower BF, BV, and PMB with higher MTT. The perfusion values of GITB lie between active and inactive CD. This is because a mixed picture is observed in GITB, wherein mild hyper-vascularity is observed in ulcers and endarteritis is observed in submucosal vessels.

This explanation of the perfusion trends is also supported by a study comparing active and inactive CD by dynamic MRI, in which the type I signal intensity curve (early upslope with a late plateau) was observed in 100% of active CD cases and the type II signal intensity curve (slow contrast material wash-in with late wash-out) was observed in 100% of inactive CD cases [30].

Our study also found an excellent correlation between the two radiologists. This proves that perfusion CT does not suffer from significant interobserver variability, which has also been validated by previous studies [11,32].

Finally, there may be concerns regarding the radiation dose of perfusion CT. The radiation dose of perfusion CT in our study is significantly less than in other studies that have attempted perfusion CT. Goh et al. conducted a study for the evaluation of radiation dose delivered in volumetric helical perfusion CT of the thorax, abdomen, or pelvis [33]. They reported a mean CTDI volume of 96.2 mGy (range: 32.3 to 169.4 mGy) and a mean DLP of 1288.8 mGy.cm (range: 648 to 2456 mGy.cm). Another study by Sitek et al. showed a mean radiation dose of 7.75 mSv [13]. This reduction in the radiation dose in our study is due to the fact that we acquired only a limited scan for perfusion. This was possible as the NCCT was performed before the perfusion scan for localization of the ileocaecal junction, so that the actual perfusion scan itself could be performed for a small region of interest. In addition, the CT scanning parameters were also adjusted in such a way that the radiation dose was minimized without compromising the diagnostic quality. We set the scanning parameters for perfusion CT to 70 kVp and 90 mAs, which is significantly less than those used in other studies. Thus, if the scanning parameters are optimized, we can reduce the radiation dose of perfusion CT, as depicted in our study.

Our study had a few limitations. First, we had a relatively small sample size. However, as this is the first study to evaluate perfusion CT in the differentiation of GITB and CD, it is highly relevant in the current scenario. Another limitation of the study was that physiological factors such as post-prandial status and atherosclerotic changes could affect vascular measurements. However, all patients who underwent perfusion CT had the same preparation before CT. None of them demonstrated CT features of significant stenosis of the superior or inferior mesenteric arteries.

## 5. Conclusions

Our study shows that perfusion CT has high sensitivity, specificity, and reproducibility in differentiating GITB from CD, as well as active from inactive CD, and may play a critical role in guiding management. Future studies on larger patient populations should try to confirm the performance of perfusion CT for the purpose of discriminating between these entities.

## Figures and Tables

**Table 1:** Mean perfusion CT parameters in the three groups.

Parameters	Inactive Crohn’s Disease (n = 9)	Gastrointestinal Tuberculosis (n = 11)	Active Crohn’s Disease (n = 6)	*p*
**Blood Flow** (mL/100 mL/min)	54.25 ± 9.15	89.49 ± 15.73	295.60 ± 21.92	0.001
**Blood Volume** (mL/100 mL)	9.03 ± 2.00	10.85 ± 3.74	10.54 ± 0.81	0.417
**Mean Transit Time** (s)	11.89 ± 2.33	8.76 ± 2.57	5.23 ± 1.95	0.001
**Permeability** (mL/100 mL/min)	11.55 ± 3.28	21.27 ± 6.43	65.97 ± 5.32	0.001

**Table 2:** Diagnostic performance of perfusion CT parameters between groups.

Parameters	Sensitivity (95% CI)	Specificity (95% CI)	PPV (95% CI)	NPV (95% CI)	Diagnostic Accuracy (95% CI)	AUC
**Gastrointestinal tuberculosis vs. Crohn’s disease**						
**Blood Flow** (mL/100 mL/min) (cut-off: 64.64 mL/100 mL/min)	69.2% (39–91)	100.0% (69–100)	100.0% (66–100)	71.4% (42–92)	82.6% (61–95)	0.692
**Blood Volume** (mL/100 mL) (cut-off: 12.68 mL/100 mL))	100.0% (75–100)	30.0% (7–65)	65.0% (41–85)	100.0% (29–100)	69.6% (47–87)	0.558
**Mean Transit Time** (s) (cut-off: 9.51 s)	61.5% (32–86)	70.0% (35–93)	72.7% (39–94)	58.3% (28–85)	65.2% (43–84)	0.600
**Permeability** (mL/100 mL/min) (cut-off: 13.9 mL/100 mL/min)	61.5% (32–86)	90.0% (55–100)	88.9% (52–100)	64.3% (35–87)	73.9% (52–90)	0.623
**Gastrointestinal tuberculosis vs. active Crohn’s disease**						
**Blood Flow** (mL/100 mL/min) (cut-off: 268.5 mL/100 mL/min)	100.0% (40–100)	100.0% (69–100)	100.0% (40–100)	100.0% (69–100)	100.0% (77–100)	1
**Blood Volume** (mL/100 mL) (cut-off: 9.8 mL/100 mL)	100.0% (40–100)	50.0% (19–81)	44.4% (14–79)	100.0% (48–100)	64.3% (35–87)	0.562
**Mean Transit Time** (s) (cut-off: 7.2 s)	100.0% (40–100)	70.0% (35–93)	57.1% (18–90)	100.0% (59–100)	78.6% (49–95)	0.875
**Permeability** (mL/100 mL/min) (cut-off: 59.1 mL/100 mL/min)	100.0% (40–100)	100.0% (69–100)	100.0% (40–100)	100.0% (69–100)	100.0% (77–100)	1
**Gastrointestinal tuberculosis vs. inactive Crohn’s disease**						
**Blood Flow** (mL/100 mL/min) (cut-off: 65.95 mL/100 mL/min)	100.0% (69–100)	100.0% (66–100)	100.0% (69–100)	100.0% (66–100)	100.0% (82–100)	1
**Blood Volume** (mL/100 mL) (cut-off: 12.79 mL/100 mL)	30.0% (7–65)	100.0% (66–100)	100.0% (29–100)	56.2% (30–80)	63.2% (38–84)	0.611
**Mean Transit Time** (s) (cut-off: 8.5 s)	70.0% (35–93)	88.9% (52–100)	87.5% (47–100)	72.7% (39–94)	78.9% (54–94)	0.811
**Permeability** (mL/100 mL/min) (cut-off: 18.81 mL/100 mL/min)	80.0% (44–97)	100.0% (66–100)	100.0% (63–100)	81.8% (48–98)	89.5% (67–99)	0.900
**Active vs. inactive Crohn’s disease**						
**Blood Flow** (mL/100 mL/min) (cut-off: 268.5 mL/100 mL/min)	100.0% (40–100)	100.0% (66–100)	100.0% (40–100)	100.0% (66–100)	100.0% (75–100)	1
**Blood Volume** (mL/100 mL) (cut-off: 9.57 mL/100 mL)	100.0% (40–100)	66.7% (30–93)	57.1% (18–90)	100.0% (54–100)	76.9% (46–95)	0.722
**Mean Transit Time** (s) (cut-off: 7.2 s)	100.0% (40–100)	100.0% (66–100)	100.0% (40–100)	100.0% (66–100)	100.0% (75–100)	1
**Permeability** (mL/100 mL/min) (cut-off: 43.54 mL/100 mL/min)	100.0% (40–100)	100.0% (66–100)	100.0% (40–100)	100.0% (66–100)	100.0% (75–100)	1

## Data Availability

The datasets generated during and/or analyzed during the current study are available from the corresponding author upon reasonable request.
